# You Can’t Always Blame the Chemo: A Rare Case of Spontaneous Tumor Lysis Syndrome in a Patient with Invasive Ductal Cell Carcinoma of the Breast

**DOI:** 10.7759/cureus.6186

**Published:** 2019-11-18

**Authors:** Meghana Parsi, Maitreyee Rai, Christina Clay

**Affiliations:** 1 Internal Medicine, Crozer-Chester Medical Center, Upland, USA; 2 Internal Medicine, Crozer Chester Medical Center, Upland, USA; 3 Hematology Oncology, Crozer Chester Medical Center, Upland, USA

**Keywords:** spontaneous tumor lysis, spontaneous tumor lysis syndrome (stls), oncologic emergency, tumor lysis syndrome (tls)

## Abstract

Tumor lysis syndrome (TLS) is an oncologic emergency characterized by metabolic and electrolyte abnormalities, observed during the destruction of tumor cells. While it is commonly seen during cytotoxic treatment of hematologic malignancies, it is rarely seen or suspected in solid tumors. The incidence of spontaneous tumor lysis (before cancer treatment) in solid malignancies is even rarer. Herein, we present the case of a spontaneous tumor lysis syndrome (STLS) in a woman who presented with chest pain and was found to have metastatic ductal cell carcinoma of the breast. She presented with acute renal failure and demonstrated all laboratory derangements consistent with TLS, despite not being on chemotherapy. Fortunately, her clinical status improved with prompt treatment, but the long-term effects of TLS can be fatal if not recognized and managed immediately. This case highlights that early recognition and appropriate treatment can be lifesaving. Furthermore, it demonstrates the importance of maintaining a high clinical suspicion in all patients with malignancy, whether hematologic or solid, of the possibility of TLS, even in the absence of chemotherapy.

## Introduction

Tumor lysis syndrome (TLS) is a well-known, potentially fatal oncologic emergency. The majority of cases occur in patients who are undergoing chemotherapy or radiation treatment for hematologic cancers. Spontaneous tumor lysis syndrome (STLS), however, is a rare occurrence prior to the onset of cytotoxic therapy, especially in patients with solid cancers. Literature review revealed only 33 other cases of STLS in solid organ malignancies, dating as far back as 1977 [1]. To our knowledge, there are less than 10 documented cases of STLS in breast cancer. TLS is an oncologic emergency, and all physicians must be aware of the possibility of STLS in patients with a solid tumor, especially when they have extensive tumor burden. We present herein the case of STLS in a female patient who presented with extensive metastatic breast cancer.

## Case presentation

A 36-year-old Caucasian female with no significant past medical history presented to the Emergency Department (ED) with a several month history of shortness of breath, pleuritic chest pain, and nonproductive cough. Two weeks before this admission, she presented to the hospital after a mass in her left breast had eroded and broke through her skin. A core biopsy at that time had confirmed an estrogen-receptor-positive (ER +), progesterone receptor-positive (PR +), and human epidermal growth factor receptor-2 positive (HER2 +) Grade 4 left invasive ductal cell carcinoma of the left breast with perineural and lymphatic invasion, for which she had not yet initiated treatment. The patient presented to the ED due to worsening exertional shortness of breath and severe, non-radiating, pleuritic chest pain.

There was no surgical history and family history was significant for a history of prostate cancer in her uncle. Social history was negative for tobacco use, ethanol use, or illicit drug use. 

**Figure 1:**
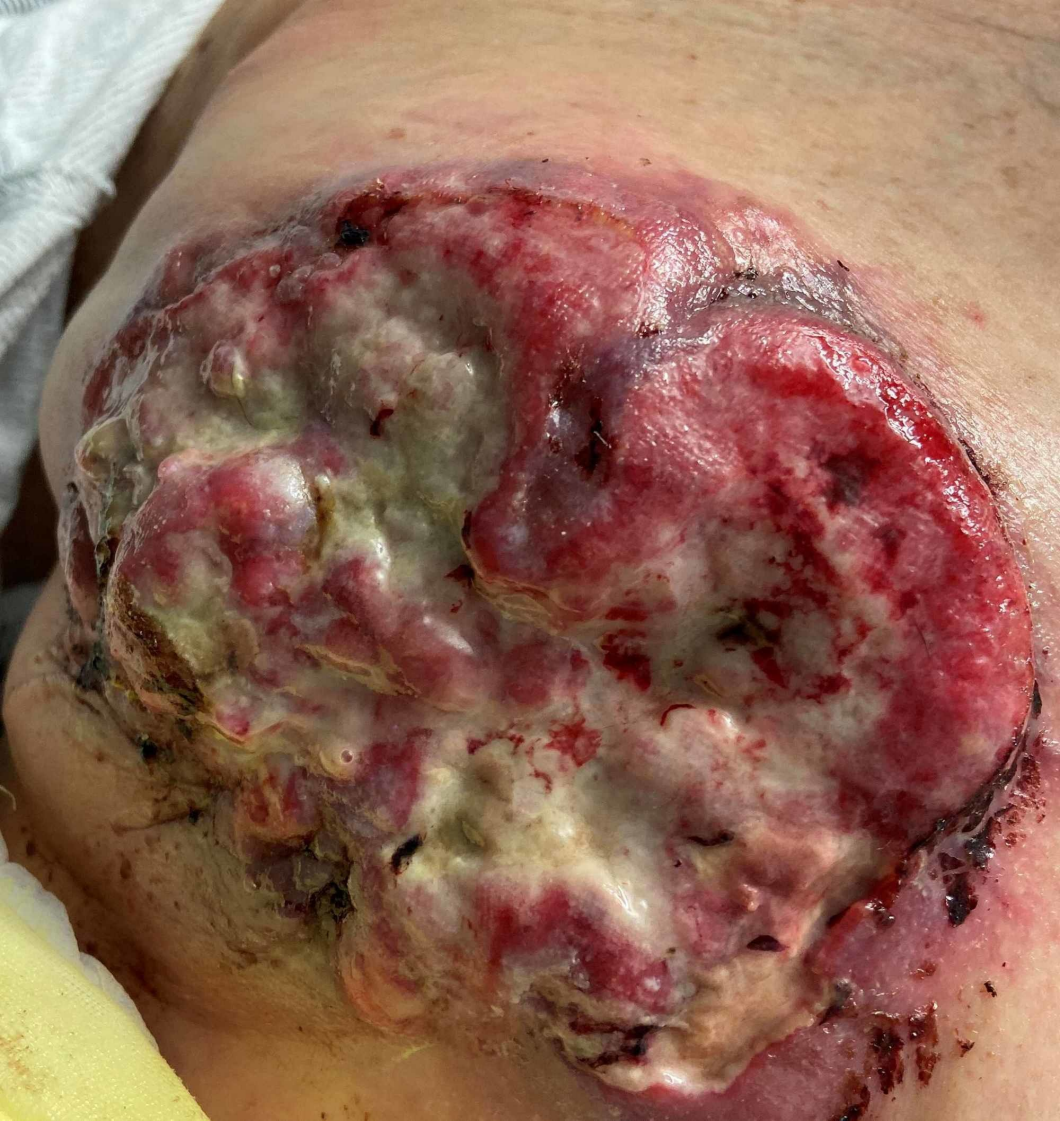
Imaging of a fungating left breast lesion with irregular margins and overlying necrotic and purulent discharge.

On exam, she was lethargic, tired, tachycardic to the 140s, tachypneic, febrile at 101° F, and hypoxic to 87% on room air, requiring oxygen via nasal cannula. The cardiovascular, pulmonary, and neurological examinations were grossly normal. The entire left breast was firm and had extensive skin thickening with a reddish discoloration. Overlying erythema, necrotic tissue, and foul-smelling purulent discharge were present (Figure 1). There was significant left axillary lymphadenopathy.

Significant laboratory values drawn on the day of admission are listed below in Table 1.

**Table 1:** Significant Laboratory Values on the Day of Admission

Parameter (Normal Range)	Admission Labs
Hemoglobin (14 - 18)	10 g/dL
White blood cell (WBC) (4.8 - 10.8)	20,000 cells/mcl with 65% neutrophils
Potassium (3.6 - 5.0)	6.5 mEq/L
Phosphorus (2.7 - 4.5 mg/dL)	4.7 mg/dL
Creatinine (0.57 - 1.11)	1.4 mg/dL
Uric Acid (2.4 - 5.7 mg/dL)	13.4 mg/dL
Total bilirubin (0.1 - 1.2)	2.3 mg/dL
Serum aspartate aminotransferase (AST) (13 - 40)	57 U/L
Alanine aminotransferase (ALT) (10 - 59)	215 U/L
Alkaline phosphatase (38 - 126)	315 U/L
Lactic acid (4.5 - 8.0)	11 mmol/L
Lactate dehydrogenase (LDH) (140 - 271)	937 U/L
C-reactive protein (CRP) (< 0.5 mg/dL)	> 300 mg/dL
Procalcitonin (< 0.5 ng/mL)	17 ng/mL
Carcinoembryonic antigen (CEA) (0 - 3 ng/mL)	249 ng/mL
Carbohydrate antigen (CA) 19-9 (< 34 U/mL)	248 U/mL

An electrocardiogram was nonischemic in nature and troponins were nondetectable. A Doppler ultrasound of the bilateral lower extremities was negative for deep venous thrombosis (DVT). 

**Figure 2:**
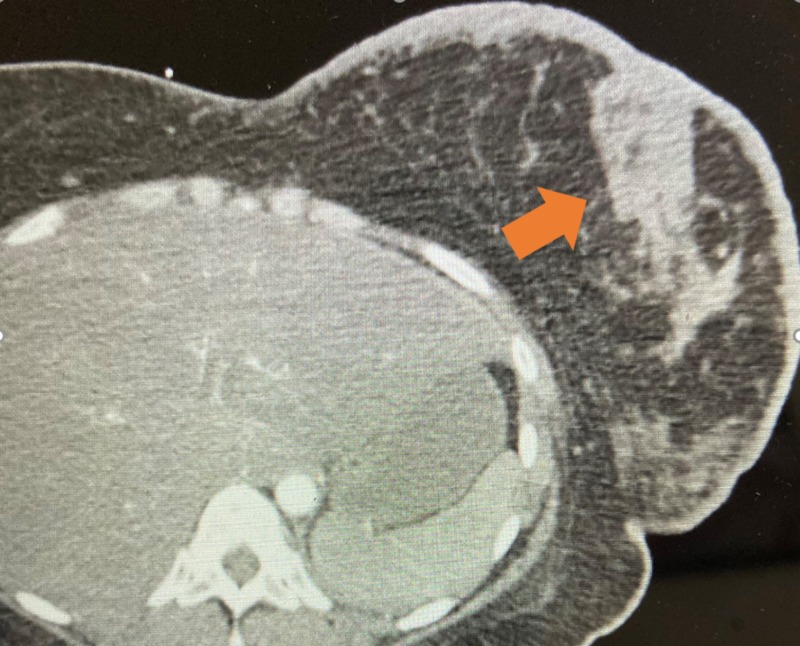
Computed tomography of the chest An abnormal soft tissue density within the left breast was noted (orange arrow), along with additional abnormal skin thickening of the left breast.

A computed tomogram (CT) scan of the chest with contrast did not reveal a pulmonary embolism but did reveal bilateral pulmonary nodules, basal consolidation, and bilateral axillary, left supraclavicular, and hilar adenopathy. There was abnormal skin thickening of the left breast, along with a 4.2 x 1.9 cm mass-like thickening involving the left pectoralis muscle (Figure 2). A CT of the abdomen and pelvis with contrast showed diffuse hepatic masses concerning for metastases. The patient was treated with antibiotics for possible pneumonia.

**Figure 3:**
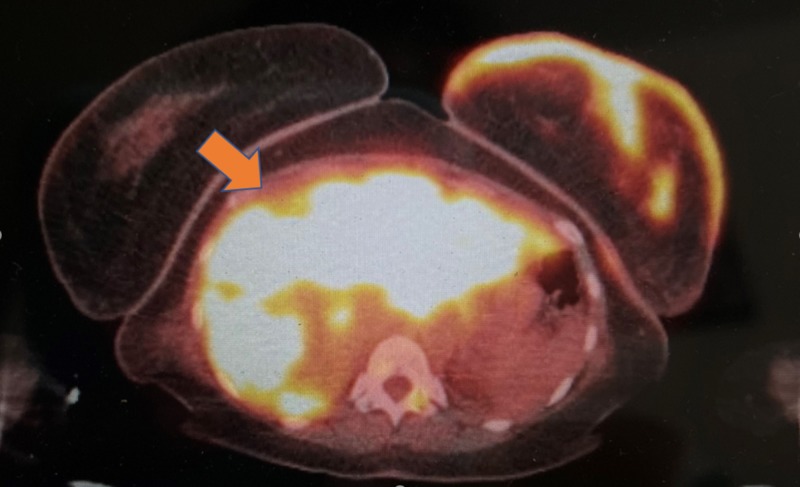
18-FDG PET/CT scan (radiation exposure 5.76 mGy) Innumerable, ill-defined hepatic lesions concerning for metastasis (orange-yellow) (intense uptake with SUV 19.8) Also illustrated is an intense uptake in the left breast (SUV 10.6). 18-FDG PET/CT: F-18 fluorodeoxyglucose positron emission tomography/computerized tomography; SUV: standardized uptake value

Mammography confirmed a poorly defined a 6 - 7 cm mass extending into the left breast with a Breast Imaging-Reporting and Data System (BI-RADS®) assessment category of 5. F-18 fluorodeoxyglucose positron emission tomography and computerized tomography (FDG PET/CT) scan revealed an abnormal uptake in the T10 vertebra, along with diffusely intense uptake in the liver, left pectoralis minor muscle, left supraclavicular, bilateral hilar, and axillary and retroperitoneal lymph nodes, all concerning for metastasis (Figure 3).

At this time, she was found to be in tumor lysis with a lactate dehydrogenase (LDH) level of 937 U/L, uric acid 13.4 mg/dL, potassium 6.5 mEq/L, phosphorus 4.7 mg/dL, and creatinine 1.4 mg/dL. An ultrasound of the kidneys was negative for obstructive uropathy. Aggressive supportive measurements with high-flow intravenous fluids and rasburicase were initiated. With the eventual resolution of her pneumonia and improvement of renal function, allopurinol was initiated for prophylaxis. With clinical and laboratory improvement, one dose of chemotherapy, including an HER-2 targeting agent, was administered before she was discharged for further outpatient management.

## Discussion

Hematologic malignancies are known to precipitate tumor lysis and these include highly aggressive lymphomas (particularly Burkitt’s lymphoma) and T-cell acute lymphoblastic leukemia (T-ALL). The incidence rate is 3% - 7% in leukemia, and 4% - 11% in lymphomas [2].

Cases of TLS are rarely reported in nonhematological solid tumors, with STLS in nonhematological solid tumors being even rarer. In fact, STLS constitutes only 15% of all TLS cases [3]. Upon review of the literature, the first case of STLS in a solid nonhematological malignancy was reported in 1977 [4]. Since then, there has been a total of only 133 cases of TLS in solid tumors, with 33 of them occurring spontaneously [5-7]. Of these, STLS occurred in four cases of breast cancer [6-9].

We believe that our case represents the fifth case of STLS in metastatic breast cancer reported in the literature.

The pathogenesis of TLS involves rapid lysis of the tumor cells with a massive release of intracellular contents into the bloodstream, with the initiation of effective cytotoxic chemotherapy, radiation therapy, or glucocorticoid treatment. This results in hyperkalemia, hyperphosphatemia, hypocalcemia (secondary to the precipitation of calcium with phosphate), and hyperuricemia (product of nucleic acid metabolism). The precipitation of calcium phosphate, xanthine, and uric acid crystals in the acidic milieu of the renal tubules leads to inflammation, obstruction, and development of acute kidney injury. Calcium phosphate can precipitate in other organs leading to ectopic calcification, such as in the cardiac conduction system, leading to the development of serious arrhythmias. These electrolyte and metabolic derangements lead to the development of multi-organ effects manifesting as nausea, vomiting, diarrhea, muscle spasms, tetany, seizures, syncope, and sudden death in severe cases [10].

The development of the above metabolic derangements in STLS is believed to occur due to a rapidly occurring tumor outgrowing its blood supply, leading to necrosis and the release of intracellular contents. In contrast to TLS after chemotherapy, patients with STLS frequently have hyperuricemia without an associated hyperphosphatemia. The possible explanation could be due to reutilization of the phosphate during the synthesis of new tumor cells [11]. Certain tumor-related factors pose an elevated risk for the development of TLS, as follows: bulky tumor, high proliferation rates, LDH > 1500, white blood cell (WBC) count > 25,000, distant metastasis with extensive bone marrow involvement, and sensitivity to chemotherapy. Other patient-related factors include preexisting kidney disease, oliguria, and volume depletion [12].

In our case, the patient already had extensive tumor burden with spread to multiple organs, including the liver. The study conducted by Harmon et al. evaluated 132 patients with solid tumors who developed TLS. The study concluded that patients with the presence of liver metastasis at the time of acute TLS had a higher mortality rate (65% of the patients with liver metastasis compared to only 26% without liver metastasis) [7].

One of the earliest classifications of TLS was by Hande and Garrow in 1993, classifying TLS into laboratory TLS (LTLS) or clinical TLS (CTLS) [13]. In 2004, the Cairo-Bishop definition was proposed and is commonly used for the diagnosis of TLS [14]. It defines LTLS as the presence of at least two of the four abnormal lab values, as listed in Table 2, that develop within three days before or seven days after anti-cancer therapy in the setting of adequate hydration and use of hypouricemic agents.

**Table 2:** Laboratory Diagnosis of Tumor Lysis Syndrome (TLS)

Cairo-Bishop Laboratory TLS Diagnostic Criteria	
Electrolyte or Metabolite	Value
Potassium	≥ 6 mEq/L or 25% increase from baseline
Phosphate	≥ 4.5 mg/dL (adults) or 25% increase from baseline
Uric Acid	≥ 8 mg/dL or 25% increase from baseline
Calcium	< 7 mg/dL or 25% decrease from baseline

CTLS is defined as LTLS when seen along with any one or more of the following clinical features, as illustrated in Table 3 [14]. Our patient met both criteria with simultaneous elevated uric acid, potassium, phosphorus, and creatinine. 

**Table 3:** Clinical Diagnosis of Tumor Lysis Syndrome (TLS)

Cairo-Bishop Clinical TLS Diagnostic Criteria
Creatinine > 1.5x the upper limit of age-adjusted range level
Cardiac dysrhythmia or sudden death
Seizure
Oliguria (< 0.5 ml/kg/h for 6 hrs)

Guidelines suggest aggressive fluid resuscitation when it comes to managing tumor lysis. Adequate hydration is vital to allow for a good flow of urine that can dispose of uric acid, potassium, and phosphate, preventing their precipitation. 

The hypouricemic agent, allopurinol, works by inhibition of xanthine oxidase (XO), the enzyme which prevents the formation of uric acid from purine metabolites. This leads to the accumulation of xanthine and hypoxanthine, which are less soluble than uric acid.

Allopurinol, however, is only effective in preventing the formation of uric acid and not in removing already formed uric acid. Rasburicase, a recombinant uricosuric agent, is used in the treatment of TLS by converting uric acid to allantoin, which is soluble. It is a Federal Drug Administration (FDA)-approved medication for treatment in patients who are receiving chemotherapy and are expected to have elevations in serum uric acid levels [15]. Hemodialysis is an option for patients with refractory electrolyte derangements or severe renal failure.

Prognosis in tumor lysis syndrome depends on the characteristics of the underlying malignancy. The mortality rate in patients with hematological malignancies is approximately 15%, with that rate increasing to 36% in patients with a solid tumor. STLS has a mortality rate of 58% [16]. Delayed recognition leading to delayed initiation of prophylaxis/treatment is at the heart of the problem. Hsu et al. proposed that STLS is, in fact, an underestimated incidence [17]. Their study evaluated 926 patients with renal failure and proposed the diagnosis of STLS on the basis of hyperuricemia, elevated LDH, and biopsy-proven malignancy. STLS was found in only 1.08% of these patients. The prognosis is grim in patients who survive the primary episode of tumor lysis. The long-term effects of renal failure, as a consequence of TLS, may inhibit further chemotherapy, thus increasing morbidity and mortality.

## Conclusions

Clinicians must be extra attentive to their patients’ risk factors (intrinsic or extrinsic) that may put them at increased risk for TLS. In patients with bulky disease or at high risk, physicians must consider prophylactic therapy. For patients with a solid malignancy, STLS must be kept in the differential if patients present with electrolyte abnormalities or renal failure, even in the absence of cytotoxic therapy. Fortunately, our patient's clinical status improved and she was able to be discharged with outpatient treatment. However, our case highlights the importance of increased awareness and appropriate education to prevent this life-threatening disease. 
